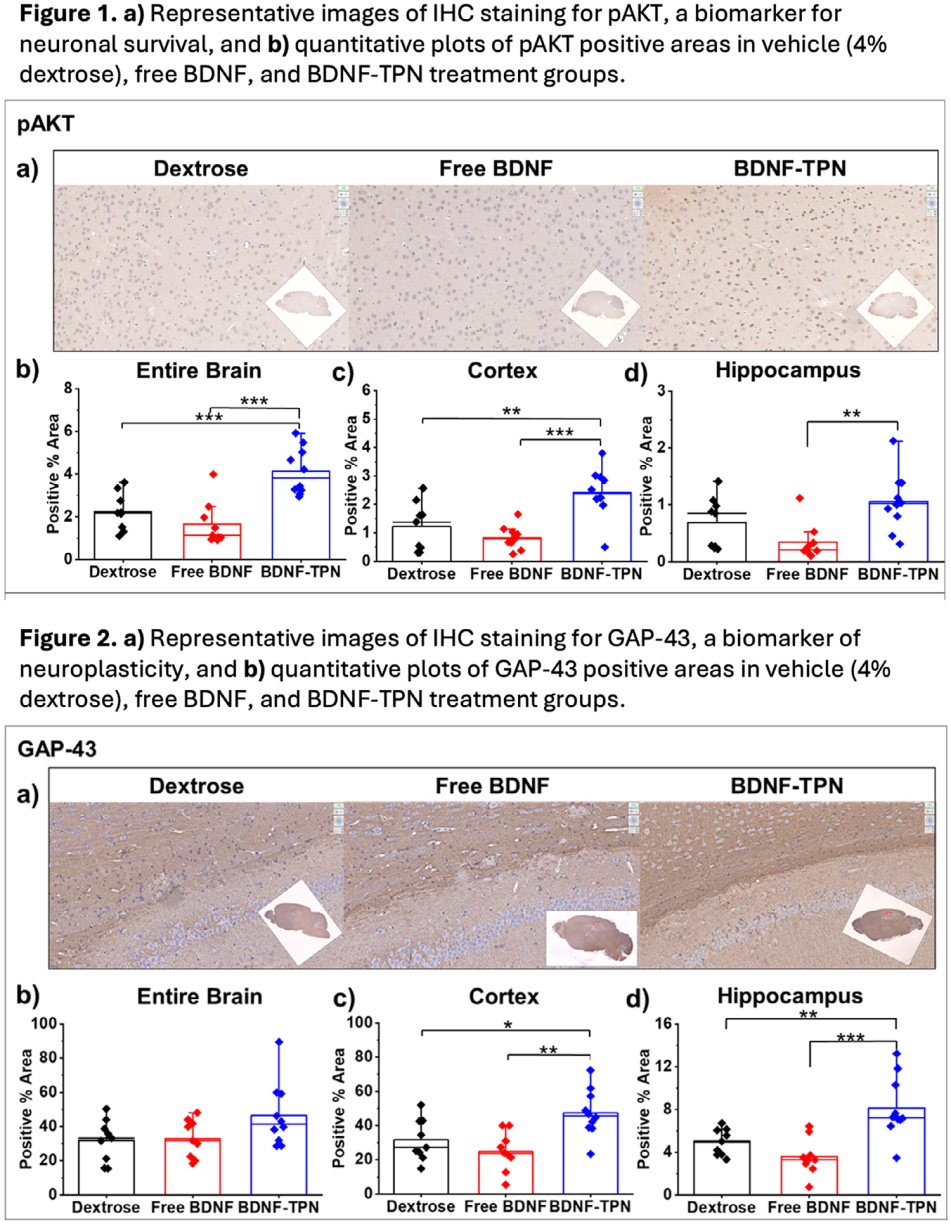# A Novel Brain‐Penetrable Nanocarrier Delivers Brain‐derived Neurotrophic Factor for Treatment of Alzheimer’s Disease

**DOI:** 10.1002/alz70859_103999

**Published:** 2025-12-26

**Authors:** Xiao Yu Wu, Lily Yi Li, Elliya Park, Chunsheng He, Azhar Z. Abbasi, Taksim Ahmed, Paul E Fraser, Andrew M. Rauth, Jeffrey T. Henderson

**Affiliations:** ^1^ University of Toronto, Toronto, ON Canada

## Abstract

**Background:**

Up to date, Alzheimer's disease (AD) has very limited disease‐modifying treatment. With respect to neuroprotection, brain‐derived neurotrophic factor (BDNF) has been shown to promote the survival and synaptic plasticity of glutamatergic and GABAergic neurons in brain regions associated with cognitive and emotive decline relevant to AD. However, the poor blood‐brain barrier (BBB) permeability and pharmacokinetic properties of BDNF limit its utilization as a neuroprotective treatment. Herein we aim to design a new BDNF nanocarrier system using a novel BBB‐permeable terpolymer (BDNF‐TPN) and investigate its neuroprotective effect in neurones and AD mice.

**Method:**

Bioactivity of the BDNF‐TPN was first evaluated in vitro using the SH‐SY5Y cell differentiation assay. The protective effects of BDNF‐TPN on primary murine hippocampal neurons were assessed following exposure to Aβ. The BDNF delivery and expression in the brain following IV injection were examined by ELISA and confocal microscopy. The biodistribution and safety were evaluated via hematologic, clinical biochemical, immunotoxicity and histology tests in APP transgenic TgCRND8 AD mice and CD‐1 mice. The effects of the treatment were evaluated in AD mice via immunohistochemistry, ELISA and behavioral test after 4‐week IV treatment (weekly, 1 mg BDNF/kg b.w.).

**Result:**

The BDNF‐TPN maintained BDNF bioactivity and rescued Aβ42 toxified primary neurons in vitro. Biomarker studies demonstrated BDNF‐mediated neuroprotective signaling in transgenic mice following IV treatment using BDNF‐TPN. Compared to free BDNF, BDNF‐TPN significantly reduced reactive microglia and astrocytes and apoptosis of neurons. The pAKT level increased more than 2‐fold in BDNF‐TPN‐treated group compared to vehicle and free BDNF treated groups. Synaptophysin, a marker for synaptic plasticity and integrity, was profoundly increased. The improved hippocampal‐dependent contextual learning in the AD mice was observed. There was not detectable toxicity following the BDNF‐TPN treatment.

**Conclusion:**

Our findings suggest BDNF‐TPN is a promising treatment for reducing neuroinflammation, apoptosis and programmed cell death in AD mouse brains, while improving synaptic plasticity and cognitive function.

**Reference**

1. Zhang W, et al. *Sig Transduct Target Ther* 2023, 8, 267.

2. He C, et al. *Nano Today*. 2020;35:100965.

3. Park E, et al. *Advanced Science*. 2023, 10(12):2207238.

4. Park E, et al. *Biomaterials*. 2025 Jan 24:123142.